# Impact Of Sex On Clinical Characteristics And In-Hospital Outcomes In A Multi-Ethnic Southeast Asian Population Of Patients Hospitalized For Acute Heart Failure

**DOI:** 10.7603/s40602-014-0008-y

**Published:** 2014-05-24

**Authors:** Carolyn S.P. Lam, Peter Chang, Shaw Yang Chia, Ling Ling Sim, Fei Gao, Fong Ling Lee, Ping Chai, Raymond Ching-Chiew Wong, Swee Chong Seow, Gerard Kui Toh Leong, Poh Shuan Daniel Yeo, David Sim, Terrance Chua, Bernard W.K. Kwok

**Affiliations:** 1 National University Health System, Cardiac Department, 1E Kent Ridge Road, 119228 Singapore, Singapore; 2 National Heart Centre Singapore, Department of Cardiology, Singapore, Singapore; 3 Khoo Teck Puat Hospital, Department of Cardiology, Singapore, Singapore; 4 Changi General Hospital, Department of Cardiology, Singapore, Singapore; 5 Tan Tock Seng Hospital, Department of Cardiology, Singapore, Singapore; 6 Singapore Cardiac Data Bank, Apex Heart Clinic, Gleneagles Hospital, Singapore, Singapore, Singapore

**Keywords:** Congestive heart failure, Sex, Characteristics, Treatment

## Abstract

**Objectives:**

To study sex differences in clinical characteristics and outcomes among multi-ethnic Southeast Asian patients with hospitalized heart failure (HHF).

**Background:**

HHF is an important public health problem affecting man and women globally. Reports from Western populations suggest striking sex differences in risk factors and outcomes in HHF. However, this has not been studied in a multi-ethnic Asian population.

**Methods:**

Using the population-based resources of the Singapore Cardiac Data Bank, we studied 5,703 consecutive cases of HHF admitted across hospitals in the Southeast Asian nation of Singapore from 1st January, 2008 through 31st December, 2009.

**Results:**

Women accounted for 46% of total admissions and were characterized by older age (73 vs. 67 years; p<0.001), higher prevalence of hypertension (78.6 vs. 72.1%; p<0.001) or atrial fibrillation (22.2 vs. 18.1%; p<0.001), and lower prevalence of coronary artery disease (33.8 vs. 41.0%; p<0.001) or prior myocardial infarction (14.9 vs. 19.8%; p<0.001). Women were more likely than men to have HHF with preserved ejection fraction (42.5% versus 20.8%, p < 0.001). Women were less likely than men to receive evidencebased therapies at discharge, both in the overall group and in the sub-group with reduced ejection fraction. Women had longer lengths of stay (5.6 vs. 5.1 days; p<0.001) but similar in-hospital mortality and one-year rehospitalization rates compared to men. Independent predictors of mortality or rehospitalization in both men and women included prior myocardial infarction and reduced ejection fraction. Among women alone, additional independent predictors were renal impairment, atrial fibrillation, and diabetes. Prescription of beta-blockers and ACE-inhibitors at discharge was associated with better outcomes.

**Conclusion:**

Among multi-ethnic Asian patients with HHF, there are important sex differences in clinical characteristics and prognostic factors. These data may inform sex-specific strategies to improve outcomes of HHF in Southeast Asians.

## Introduction

Hospitalized heart failure (HHF) is a disease that knows no geographic, gender, or socioeconomic boundaries. It is a leading cause of morbidity and mortality worldwide, and is projected to rise with increasing longevity of populations. The largest increase in the global burden of cardiovascular disease is occurring in the rapidly aging region of Southeast Asia, where men and women have long differed in socioeconomic status, health beliefs and access to medical care^1^.

Recent heart failure studies have highlighted important geographic differences in heart failure patients as well as in heart failure etiologies between women and men^2^.These studies have demonstrated striking differences in the prevalence, clinical presentation, risk factors, treatment and prognosis between men and women with HHF in Middle Eastern^3^,^4^,American^5^, and European populations^6^. Recent meta-analyses confirmed gender to be a key determinant of outcomes in HHF^7^ and further suggested the presence of gender differences in treatment^8^,^9^. While prior studies have highlighted fundamental sex differences in HHF, most of these data were derived in Western populations. In contrast, data are scarce regarding sex differences in HHF among Asian populations.

Accordingly, we aimed to study sex differences in the clinical characteristics and outcomes of HHF in the multi-ethnic Southeast Asian population of Singapore. The availability of a nation-wide HHF Registry (part of the Singapore Cardiac Data Bank) provided a unique population-based resource to systematically investigate sex differences in a large, consecutively recruited sample of Southeast Asian patients with HHF.

### Methods

Study Population: The Heart Failure Registry of the Singapore Cardiac Data Bank is a prospective study of consecutive patients admitted with HHF across all public institutions in Singapore. These public institutions together serve >80% of the population of Singapore. The Singapore population consists of Chinese, Malay, and Indian ethnicities.

Data was systematically collected in each patient from initial point of care to discharge, and included detailed information such as demographics, admission data, medical history, initial evaluation, laboratory results, procedures performed, medications and discharge summary. For the purpose of this registry, HHF was defined as either new-onset HF with acute decompensation or chronic HF with acute decompensation requiring hospitalization. Patients were included if they were at least 21 years of age at the time of admission, and received a primary hospital discharge diagnosis of HF. Patients were excluded if HF was only a comorbid condition and not the principal focus of diagnosis or treatment during hospitalization. The outcomes of interest in this analysis were length of stay, in-hospital mortality, and rehospitalization. Follow-up was complete and death data were ascertained from the National Registry of births and deaths. All patients provided informed consent. The study was approved by the Institutional Research Boards of all participating hospitals.

### Statistical Analysis

Variables were included in statistical analyses only if < 10% of the collected data was missing. For comparison of baseline characteristics between genders, Chi-square test was used for categorical variables and Wilcoxon rank-sum test used for continuous variables. For assessment of predictors of mortality and re-hospitalization, hazard ratios and 95% CI were calculated using logistic regression, in the entire group and stratified by sex. Preserved LVEF was defined as LVEF greater or equal to 50%. Anemia was defined as a hemoglobin concentration of less than or equal to 12 g/dL in women and 13g/ dL in men. Variables with a significance of <0.05 in univariate analysis were entered in the multivariate model. In multivariable modeling both stepwise and backward variable selection techniques were used with a p-value of 0.05 as criteria for both entering and remaining in the model. Hazard ratios and 95% confidence intervals were presented. Cohorts of patients were analyzed both by gender as well as by LVEF. All analyses were performed using SPSS statistical software (version 18.0, SPSS Inc., Chicago, Illinois (IL).

## Results

There were a total of 8,657 cases of HHF admitted from January 2008 until December 2009. Excluding 2954 recurrent admissions, a final sample of 5,703 patients, of whom 2,635 (46.2%) were women, was analyzed in this study.

### Baseline characteristics

Baseline clinical characteristics by sex are presented in Table 1 and Figure 1. Women were on average, six years older than men (mean age 73.1 years versus 67.4 years, p < .001); were more likely to have hypertension (78.6% versus 72.1%, p <0.001), diabetes (55.7% versus 50.2%, p <0.001) or a history of atrial fibrillation or fl utter (22.2% versus 18.1%, p <0.001); and less likely to have coronary artery disease (33.8% versus 41.0%, p <0.001) or prior myocardial infarction (14.9% versus 19.8%, p <0.001), or to be a current smoker (3.7% versus 22.7%, p <0.001) compared to men. Body size comparisons were not included due to a high percentage of missing data.

At presentation (Table 1), women and men had similar clinical signs and symptoms of HF (fatigue, rales, and peripheral edema). However, women had higher mean systolic blood pressure on admission (147±30 mmHg versus 142±39 mmHg, p < 0.001), and lower median admission creatinine (99.0 umol/L versus 117.0 umol/L; p < 0.001), than men. Women had a higher prevalence of anemia than men (59.6% versus 51.2%, p < 0.001). Women were more likely than men to have HF with preserved LVEF (42.5% versus 20.8%, p < 0.001).

### Procedures and treatment

The initial point of care in both women and men was the emergency department (94.7% versus 93.8%, p=0.473) followed by admission to general ward (92.0% versus 90.2%, p=0.144). There were no sex differences in the use of renal replacement therapy, mechanical ventilation, defibrillation or cardiopulmonary resuscitation. Invasive procedures such as coronary angiography were performed less often in women than men (1.4% versus 2.8%, p< 0.001).

### Cardiovascular medication on discharge

Overall, women were less likely than men to receive ACE inhibitors or angiotensin receptor blockers (65.4% versus 71.9%, p < 0.001), aldosterone receptor antagonists (9.5% versus 15.7%, p < 0.001), loop diuretics (80.1% versus 84.1%, p < 0.001), nitrates (30.0% versus 33.5%, p < 0.001), digoxin (23.2% versus 27.5%, p < 0.001), and beta-blockers (57.8% versus 64.2%, p < 0.001) upon discharge (Table 2). Rates of contraindication or intolerance to ACE inhibitors, ARB, and betablockers were low and did not differ by sex. Subanalysis by LVEF group revealed a similar pattern of under prescription of discharge medications in women compared to men with HF with reduced LVEF. In the preserved LVEF subgroup, rates of prescription of heart failure medications were similarly low in women and men (Table 2).

### Outcomes

Mean length of stay was longer for women than men (5.62 versus 5.05 days, p< 0.001) (Table 3). There was no sex difference in in-hospital mortality (1.9% versus 1.7%, p=0.638). Adjusting for age and co-morbidities, the rate of rehospitalization at one year was similar between men and women (26.2% versus 26.3%, p=0.891). In stratified analyses by LVEF group, mean length of stay was longer for women than men in both HF with reduced LVEF as well as HF with preserved LVEF. In-hospital mortality and rate of rehospitalization at one year outcomes were similar between men and women by LVEF group.

### Univariable correlates of outcomes

Univariable predictors of mortality or rehospitalization are summarized in Table 4. Among women, significant univariable predictors of outcome included age, LVEF less than 50%, renal disease, coronary artery disease, prior myocardial infarction, diabetes, peripheral vascular disease, prior cerebrovascular event, atrial fibrillation, and anemia. Among men, significant univariable predictors of outcome were age, LVEF less than 50%, renal disease, coronary artery disease, prior myocardial infarction, peripheral vascular disease, prior cerebrovascular event, and anemia. Treatment with ACE inhibitors or ARB, beta-blockers, aspirin, and lipid-lowering medications, were associated with better outcomes in both men and women.

### Multivariable correlates of outcomes

Multivariable predictors of outcome and their hazard ratios are shown in Table 5. Among women, independent predictors of outcome included renal disease, history of prior myocardial infarction, atrial fibrillation, diabetes, and reduced LVEF. Among men, independent predictors of outcomes included prior myocardial infarction, reduced LVEF, and anemia. Treatment with beta-blockers and ACE inhibitors or ARB was associated with better outcomes in both men and women.

Left ventricular systolic dysfunction (LVEF < 50%) was present in 3946 patients (69.2%). In patients with HF and reduced LVEF, independent predictors of mortality or rehospitalization included prior myocardial infarction, prior cerebrovascular accident, diabetes, admission blood urea nitrogen, and anemia. In patients with HF and preserved LVEF, independent predictors of outcome included renal disease, prior myocardial infarction, atrial fibrillation, hypertension and admission blood urea nitrogen.

Treatment with beta-blockers and ACE inhibitors or ARB was associated with better outcomes in both HF groups.

Left ventricular systolic dysfunction (LVEF < 50%) was present in 3946 patients (69.2%). In patients with HF and reduced LVEF, independent predictors of mortality or rehospitalization included prior myocardial infarction, prior cerebrovascular accident, diabetes, admission blood urea nitrogen, and anemia. In patients with HF and preserved LVEF, independent predictors of outcome included renal disease, prior myocardial infarction, atrial fibrillation, hypertension and admission blood urea nitrogen. Treatment with beta-blockers and ACE inhibitors or ARB was associated with better outcomes in both HF groups.

## Discussion

The multi-ethnic population of Singapore and large number of patients captured in our prospective nationwide HF registry provided an opportunity to study sex differences in the clinical characteristics and outcomes of HHF in Southeast Asians. Women made up nearly half of the heart failure admissions in our Southeast Asian population. Unsurprisingly, co-morbidities of hypertension, diabetes, and anemia were prevalent in over 50% of subjects studied. Compared to men, Asian women with HHF were notably older, more often hypertensive and had higher prevalence of atrial fibrillation, diabetes and anemia, but lower prevalence of coronary artery disease.

Women were also twice as likely as men to present with HHF with preserved LVEF. Our findings are consistent with reports from European, American and Middle Eastern populations, and extend these observations to a multi-ethnic Southeast Asian population in whom data were previously lacking. These findings suggest that the underlying mechanism of heart failure in women is likely that of chronic disease where deleterious effects of age and metabolic disease state on diastolic function predominate. In contrast, the mechanism of heart failure in men is likely from coronary artery disease or ischemic heart disease resulting in myocardial necrosis and reduction of systolic function.

There were some differences between our findings and that from other HHF cohorts. The mean length of hospitalization was longer in women than men in our Asian cohort, in contrast to findings of ADHERE^8^ or EHFSII^6^. The difference in hospitalization stay could not be explained by sex differences in the level of hospital care, hospital course and procedures performed, which were similar in men and women in our cohort. Nonetheless, the inhospital mortality and rehospitalization rates were similar in women and men in our cohort, consistent with data from European and American cohorts where no gender difference was found in clinical outcomes^6^,^8^,^10^,^11^. This suggests that the regional differences in length of stay may refl ect differences in sociocultural factors and health seeking behavior of Asian women versus men, rather than differences in severity or pathophysiology of disease.^12^ Future prospective studies with longer follow-up are warranted to further elucidate potential sex differences in outcomes and underlying reasons.

Our observed sex differences in the use of evidencebased medications deserve mention. Evidence-based standard of care regimen for HF with reduced LVEF, including ACE inhibitors and beta-blockers, were prescribed in the majority of patients with HHF and reduced LVEF in our cohort. However, compared to men, women in our cohort with HHF and reduced LVEF were less likely to be prescribed ACE inhibitor/ ARB classes of medications or beta-blockers^13^-^16^.

These significant differences in prescription rates existed in spite of similar rates of known contraindications to medications in women and men. There was also a lower rate of prescription of aldosterone receptor antagonists, loop diuretics, digoxin, aspirin, and lipid-lowering medications among women compared to men in our cohort. This trend of under-treatment in women is likely multifactorial. Clinical trials and physician perception of typical heart failure patients have been more focused on men than women. A reduced LVEF has been traditionally used to defi ne systolic dysfunction, assess prognosis, and select patients for therapeutic interventions.

According to current international HF guidelines there is, to date, no evidence-proven therapy that unequivocally improves outcomes in HF with preserved LVEF^17^,^18^.The higher prevalence of HF with preserved LVEF in women and the lack of knowledge on treatment and outcome of HF with preserved LVEF may also explain the lower rates of prescription of ACE inhibitors and beta-blockers in women, although this cannot explain the persistent differences in our subgroup analysis of HF with reduced LVEF alone. Importantly, ACE inhibition and beta-blockade were associated with better outcomes in both men and women in our study. Our findings call for attention to potential sex-specific barriers and biases in prescribing practice in our population.

We also found sex differences in prognostic variables in our cohort. In particular, there was a striking overlap of renal disease and atrial fibrillation in women and in those with HF with preserved LVEF. This is consistent with findings from the large I-PRESERVE study of HF with preserved LVEF^19^, and underscores the importance of recognizing women at risk of poor outcomes, even in Asian populations.

### Study limitations

We acknowledge the inherent limitations of an observational database and retrospective analysis. Nonetheless, our registry data were comprehensively and prospectively collected, involving all public hospitals in Singapore. Our results refl ect the practice in 2008 when aldosterone inhibitors were not as widely prescribed and device therapy not as widely available in Singapore as the current period. More contemporary studies with longer follow-up are warranted.

## Conclusion

These fi rst population-based data in a multi-ethnic Southeast Asian cohort with HHF demonstrate important sex differences in clinical characteristics and risk factors. Women make up nearly half of all patients admitted for acute heart failure in Singapore. Compared to men, women with HHF are older, more often hypertensive, more likely to have atrial fibrillation and twice as likely to have preserved LVEF. On discharge, heart failure directed therapies are underutilized in women. Despite longer hospital stay in women, one-year mortality and rehospitalization rates were similar in men and women. These data may inform sex-specific strategies to improve outcomes of HHF in Southeast Asians.

Open Access: This article is distributed under the terms of the Creative Commons Attribution License (CC-BY 4.0) which permits any use, distribution, and reproduction in any medium, provided the original author(s) and the source are credited.
